# Differential sensitivity to apoptosome apparatus activation in non-small cell lung carcinoma and the lung

**DOI:** 10.3892/ijo.2014.2333

**Published:** 2014-03-10

**Authors:** ERIKA MORAVCIKOVA, EVZEN KREPELA, JAN PROCHAZKA, KAMILA BENKOVA, NORBERT PAUK

**Affiliations:** 1 Laboratories of Molecular and Cell Biology, Hospital Bulovka and Third Faculty of Medicine, Charles University in Prague; 2 Division of Pneumology, Department of Pneumology and Thoracic Surgery, Hospital Bulovka and Third Faculty of Medicine, Charles University in Prague; 3 Department of Pathology, Hospital Bulovka; 4 Institute of Biochemistry and Experimental Oncology, First Faculty of Medicine, Charles University in Prague, Prague, Czech Republic

**Keywords:** non-small cell lung carcinoma, apoptosome, Apaf-1, caspase, X-linked inhibitor of apoptosis

## Abstract

The intrinsic apoptosis pathway represents an important mechanism of stress-induced death of cancer cells. To gain insight into the functional status of the apoptosome apparatus in non-small cell lung carcinoma (NSCLC), we studied its sensitivity to activation, the assembly of apoptosome complexes and stability of their precursors, and the importance of X-linked inhibitor of apoptosis (XIAP) in the regulation of apoptosome activity, using cell-free cytosols from NSCLC cell lines and NSCLC tumours and lungs from 62 surgically treated patients. Treatment of cytosol samples with cytochrome *c* (cyt-*c*) and dATP induced proteolytic processing of procaspase-9 to caspase-9, which was followed by procaspase-3 processing to caspase-3, and by generation of caspase-3-like activity in 5 of 7 studied NSCLC cell lines. Further analysis demonstrated formation of high-M_r_ Apaf-1 complexes associated with cleaved caspase-9 in the (cyt-*c* + dATP)-responsive COLO-699 and CALU-1 cells. By contrast, in A549 cells, Apaf-1 and procaspase-9 co-eluted in the high-M_r_ fractions, indicating formation of an apoptosome complex unable of procaspase-9 processing. Thermal pre-treatment of cell-free cytosols in the absence of exogenous cyt-*c* and dATP lead to formation of Apaf-1 aggregates, unable to recruit and activate procaspase-9 in the presence of cyt-*c* and dATP, and to generate caspase-3-like activity. Further studies showed that the treatment with cyt-*c* and dATP induced a substantially higher increase of caspase-3-like activity in cytosol samples from NSCLC tumours compared to matched lungs. Tumour histology, grade and stage had no significant impact on the endogenous and the (cyt-*c* + dATP)-induced caspase-3-like activity. Upon addition into the cytosol, the XIAP-neutralizing peptides AVPIAQK and ATPFQEG only moderately heightened the (cyt-*c* + dATP)-induced caspase-3-like activity in some NSCLC tumours. Taken together, the present study provides evidence that the apoptosome apparatus is functional in the majority of NSCLCs and that its sensitivity to the (cyt-*c* + dATP)-mediated activation is often enhanced in NSCLCs compared to lungs. They also indicate that XIAP does not frequently and effectively suppress the activity of apoptosome apparatus in NSCLCs.

## Introduction

The apoptosome-driven cell death pathway plays an important role not only in embryonic development and homeostasis of postnatal tissues (1,2), but it can be also involved in pathogenesis and treatment response of cancer (3,4). The apoptosome pathway is launched upon cytochrome *c* (cyt-*c*) release from mitochondria into the cytosol (5). Cyt-*c* molecules bind to cytosolic Apaf-1 monomers containing 13 WD repeats (6,7) and induce, together with (d)ATP binding via nucleotide exchange, a conformation change of Apaf-1 monomers allowing them to oligomerize into a heptameric complex called apoptosome (8,9). Subsequent binding of procaspase-9 (PC-9) molecules to apoptosome leads to their activation via autoproteolytic processing, yielding the active apoptosome-bound cleaved caspase-9 (CS-9) (8,10–12). The active CS-9 in the holo-apoptosome then cleaves and activates the zymogens of the executioner caspase-3 (CS-3) and caspase-7 (CS-7) (8,10–14).

The processes of assembly and function of apoptosome complexes can be positively or negatively regulated by numerous factors (15,16). There is evidence that not only dysfunction of apoptosome (17–20), but also its hyperactivity (21–24) can contribute to development and progression of malignant tumours and their susceptibility to therapy. It has been reported that although numerous non-small cell lung carcinoma (NSCLC) cell lines and tumours express Apaf-1, PC-9 and procaspase-3 (PC-3) proteins in levels sufficient to launch the apoptosome pathway, their capability of the apoptosome-dependent caspase activation may be low or absent (25–28). Despite the studies of several possible regulators of apoptosome in NSCLC cells, including the inhibitor of apoptosis proteins XIAP, cIAP-1 and cIAP-2, TUCAN, Smac, and PHAPI (28–32), and the evidence of high-M_r_ apoptosome complexes incapable of PC-9 processing (33–35), the regulation of apoptosome assembly and signalling in NSCLC is still elusive.

We demonstrated previously that although the levels of PC-9 protein were comparable in NSCLC tumours and matched lungs, the expression of both Apaf-1 and PC-3 proteins was frequently upregulated and the induced activity of apoptosome apparatus tended to be higher in the tumours as compared to lungs (27). To explore further the functionality of apoptosome apparatus in NSCLC, we studied its sensitivity to activation in the cell-free cytosol from NSCLC cells and NSCLC tumours and matched lungs, the assembly of apoptosome complexes and functional stability apoptosome precursors, the impact of clinico-pathological parameters of NSCLC tumours on the level of apoptosome-generated CS-3-like activity, and the involvement of XIAP in the regulation of apoptosome activity in NSCLC tumours.

## Materials and methods

### Reagents

Most reagents used in this study were obtained from suppliers as described previously (27). Sephacryl S300HR, Gel Filtration Molecular Weight Markers (cat. no. MW-GF-1000), bovine serum albumin (BSA; cat. no. A7030), the affinity purified rabbit anti-caspase-3 and rabbit anti-Apaf-1 antibodies (cat. nos. C9598 and A8469, respectively), and the goat anti-rabbit IgG horseradish peroxidase (HRP) conjugate (cat. no. A4914), used as a secondary antibody, were from Sigma (St. Louis, MO, USA). The rabbit anti-caspase-9 antibody was from Cell Signaling (cat. no. 9502, Danvers, MA, USA). The pre-stained Precision Plus Protein Standards and Blotting-Grade Blocker (BGB) were from Bio-Rad Laboratories (Hercules, CA, USA). The peptides AVPIAQK (P1) and ATPFQEG (P2) were custom synthesized by Clonestar Peptide Services (Brno, Czech Republic).

### Cell cultures

NSCLC cell lines used in the present study were squamous cell lung carcinoma-derived cell lines CALU-1, NCI-H520 and SKMES-1, and lung adenocarcinoma-derived cell lines A549, SKLU-1, LXF-289 and COLO-699. They were obtained from the following depositories: CALU-1, SKMES-1, A549 and SKLU-1 were from the European Collection of Cell Cultures (Salisbury, UK), LXF-289 and COLO-699 were from the German Collection of Microorganisms and Cell Cultures (Braunschweig, Germany), and NCI-H520 was from the American Type Culture Collection (ATCC, Rockville, MD, USA). The cells were cultured in humidified atmosphere of 5% CO_2_ and air at 37°C in the Eagle’s minimum essential medium supplemented with 2 mM L-glutamine, 26.2 mM NaHCO_3_, 1% of a stock solution of non-essential amino acids, 10 mg/l apo-transferrin, 0.25 *μ*M Fe(NO_3_)_3_, 5% foetal bovine serum, 105 IU/l of penicillin-G and 100 mg/l of streptomycin. After reaching confluence, the cells were harvested for preparation of cell-free cytosols.

### Patients and tissues

Specimens of NSCLC tumours and matched lung parenchyma were obtained from 62 surgically treated patients (median age of 63 years, age range of 47–78 years; 41 men and 21 women; 48 smokers, 10 non-smokers and 4 ex-smokers), who did not received radiotherapy nor chemotherapy before surgery for lung cancer. The study was approved by the local institutional ethics committee and was conducted in accordance with the Declaration of Helsinki. Signed written informed consent was obtained from each patient before entry to the study. Tissue samples (∼1 g, wet mass) from non-necrotic part of the tumour and from the lung parenchyma at a site located as distantly as possible from the tumour, were excised from the resected lung lobe or lung immediately after surgery. All tissues were snap-frozen in liquid nitrogen and stored at −78°C until cell-free cytosols preparation. The histopathological classification of the tumours was done according to the World Health Organization criteria (36) and included squamous cell lung carcinoma (SQCLC; n=30, including 1, 15 and 14 tumours of grade 1, 2 and 3, respectively), lung adenocarcinoma (LAC; n=25, including 3, 8 and 11 tumours of grade 1, 2 and 3, respectively), mixed type SQCLC and LAC (n=1), large-cell lung carcinoma (LCLC; n=1), sarcomatoid lung carcinoma (SLC; n=3) and undifferentiated lung carcinoma (UNDIF; n=2). Tumour staging was performed according to the new international pTNM system (37), and included 9 and 21 tumours of stage IA and IB, respectively, 2 and 13 tumours of stage IIA and IIB, respectively, 15 and 1 tumours of stage IIIA and IIIB, respectively, and 1 tumour of stage IV.

### Preparation of cell-free cytosol samples

Unless otherwise indicated, the preparation of cell-free cytosols was performed at 0–4°C. Cells at confluence were washed and gently scraped into the Hank’s balanced salt solution containing 4.16 mM NaHCO_3_. Subsequently, cells were centrifuged at 300 × g for 10 min and the pellets were resuspended in 1/4-1/2 volume of 25 mM HEPES/NaOH, 4 mM Na_2_EDTA, 1.5 mM MgCl_2_, 10 mM KCl buffer, pH 7.4 (HEMK buffer). After gentle shaking for 30 min, the cells were disrupted using whole glass Dounce homogenizer equipped with the pestle B. To prepare tissue cytosols, samples of NSCLC tumours and lung parenchyma were disrupted using three cycles of liquid nitrogen pre-chilling in a Teflon container and pulverizing for 30 sec on Mixer Mill MM 200 (Retsch, Haan, Germany) at a speed of 30 sec^−1^. The powders were resuspended in HEMK buffer (1.5 ml per 1 g of wet tissue) and shaken on ice for 30 min. The suspensions were homogenized three times for 5 sec on a homogenizer Ultra-Turrax T25 (IKA, Staufen, Germany) at 24,000 rpm. The cell and tissue homogenates were centrifuged at 200,000 × g for 60 min and the supernatants (i.e. cytosols) were stored in small aliquots at −78°C until analysis.

### Apoptosome apparatus activation and assay of caspase-3- like activity

In order to activate the apoptosome apparatus, cytosol samples (150 *μ*l), containing 2.5 mg of total protein/ml in HEMK buffer with 5 mM D,L-dithiothreitol (DTT), were incubated with 10 *μ*M of cyt-*c* and 1 mM of dATP at 37°C for 30 min. When analysing the effect of the XIAP-neutralizing peptides AVPIAQK (P1) and ATPFQEG (P2), the peptides were added to the activation reactions in a concentration of 500 *μ*M, which was previously shown to be very effective in relieving the (cyt-*c* + dATP)-induced apoptosome activity from the XIAP-mediated inhibition (38,52). Cytosol samples incubated without cyt-*c* and dATP and without the peptides P1 and P2 served as controls. Subsequently, aliquots of the incubated cytosol samples (20 *μ*l) were used for measurement of the endogenous (E, in the controls) and the total (cyt-*c* + dATP)-induced (T) CS-3-like activity, using a kinetic enzyme assay. The incremental induced (I) CS-3-like activity was then calculated as the difference between the T and E activities (I=T-E). The enzyme reactions, carried out in a total volume of 200 *μ*l at 37°C and in duplicate, were done with 100 *μ*M of Ac-DEVD-AFC as a substrate in an assay buffer containing 50 mM HEPES/NaOH, 1.63 mM CHAPS, 1 mM Na_2_EDTA, 292 mM sucrose, 100 mM NaCl, and 5 mM DTT, pH 7.2. Further control reactions, run in parallel, were performed in the presence of a caspase inhibitor Ac-DEVD-CHO (10 *μ*M). The fluorescence of the enzymatically released 7-amino-4-trifluoromethylcoumarin (AFC) was measured on a microplate fluorometer SpectraFluor (Tecan, Salzburg, Austria). The caspase activity was calculated from the progress curves in steady state. The specific caspase activity was expressed in pkat (pmol/sec) per 1 mg of total protein.

### Gel filtration chromatography

For the study of the assembly of apoptosome complexes, samples of cell-free cytosol from COLO-699, CALU-1 and A549 cells (5 mg of total protein/ml) were incubated in HEMK buffer with 5 mM DTT in the presence or absence of cyt-*c* (10 *μ*mol/l) and dATP (1 mmol/l) at 37°C for 30 min. Subsequently, the samples were fractionated by gel filtration chromatography (GFC) on a column (70×1.6 cm) of Sephacryl S300HR at 2–4°C. The column was eluted with a buffer containing 25 mM HEPES/NaOH, 1 mM Na_2_EDTA, 1.5 mM MgCl_2_, 10 mM KCl, 1 mM DTT, and 100 mM NaCl, pH 7.2. The elution rate was 0.5 ml/min and 0.5-ml fractions were collected. Aliquots of the fractions were denatured in a sample buffer and were subjected to sodium dodecyl sulphate-polyacrylamide gel electrophoresis (SDS-PAGE) followed by immunoblotting analysis. In some experiments, the samples of cell-free cytosol without exogenous cyt-*c* and dATP, serving as a control, were kept on ice before loading onto the chromatography column. The column was calibrated with Blue Dextran 2000 and 6 different protein M_r_-markers.

### SDS-PAGE and immunoblotting

The assembly of apoptosome complexes and the proteolytic processing of PC-9 and PC-3 in the control and the (cyt-*c* + dATP)-treated cell-free cytosols were investigated by SDS-PAGE and immunoblotting using specific antibodies. Prior loading onto SDS-polyacrylamide gels, cytosol samples (2.5 or 5 mg of total protein/ml) were incubated at 37°C for the indicated times without or with cyt-*c* (10 *μ*M) and dATP (1 mM) in HEMK buffer, pH 7.4, with 5 mM DTT. Where indicated, the caspase inhibitor Ac-DEVD-CHO (1 *μ*M) was added into the incubated samples. SDS-PAGE was carried out in 16.5% T/3% C and 8% T/3% C gels, for analysis of caspases and Apaf-1, respectively, using the Tris-Tricine-SDS buffer system (39). The samples were denaturated by 5 min boiling in a sample buffer, pH 7.4, containing 2% SDS, 0.01% Serva Blue G, 50 mM Tris/HCl, 100 mM DTT, and 10% glycerol, and were gel-loaded (50 *μ*g of total protein per lane) in parallel with pre-stained protein M_r_ markers. The separated proteins were electrotransferred to Hybond-P PVDF membrane sheets (GE Healthcare, Little Chalfont, UK) with using a transfer buffer containing 48 mM Tris, 39 mM glycine, 1.3 mM SDS, 20% methanol, pH 9.2. The following immunoblotting procedure, coupled with an enhanced chemiluminescence detection system, was carried out at room temperature as follows. The membrane sheets were blocked with 5% BGB and 1% BSA in TBST buffer (20 mM Tris/HCl, 100 mM NaCl, 0.1 v/v% Tween-20, pH 7.6) for 1 h, and incubated with anti-Apaf-1 antibody (1 *μ*g/ml) or anti-caspase-3 antibody (0.5 *μ*g/ml) for 2 h or with anti-caspase-9 antibody (at a dilution of 1:1,500 or 1:500) overnight at 4°C. The primary antibodies were diluted in TBST buffer. The membranes were then extensively washed in TBST buffer, incubated with the secondary HRP-conjugated antibody (at a dilution of 1:10,000 in 5% BSA in TBST buffer) for 1 h, extensively washed in TBST buffer, and incubated with the ECL Plus Reagent (GE Healthcare) for 5 min. The chemiluminiscence signal of specific immunocomplexes was captured onto BioMax Light-1 film (Eastman Kodak, Rochester, NY, USA).

### Determination of XIAP and total protein

The levels of XIAP protein in cell-free cytosol samples were determined by sandwich ELISA as described previously (40). Total protein concentration in cell-free cytosol samples was determined by the bicinchoninic acid assay using BSA as a standard (41).

### Statistical analysis

The statistical analysis was performed with SigmaStat software (Systat Software, Point Richmond, CA, USA) and Stat200 (Biosoft, Cambridge, UK). A two-sided p-value lower than 0.05 was accepted as statistically significant difference.

## Results

**Figure 1.**
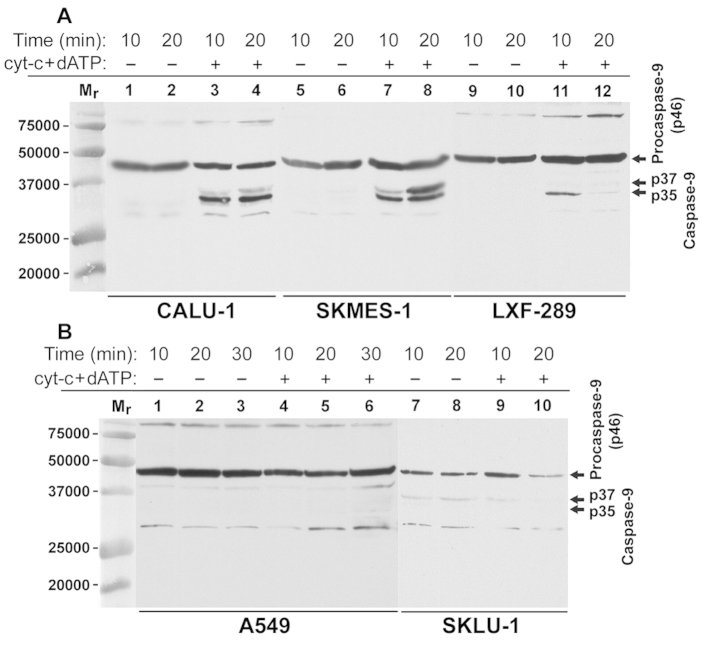
Analysis of the (cyt-*c* + dATP)-induced procaspase-9 processing in cell-free cytosols from NSCLC cell lines. (A) Incubation of cell-free cytosol samples from CALU-1, SKMES-1 and LXF-289 cells with exogenous cyt-*c* and dATP induced proteolytic processing of procaspase-9 (p46) to the p35 and p37 cleaved caspase-9 forms. (B) Procaspase-9 (p46) in cell-free cytosol samples from A549 and SKLU-1 cells was resistant to the (cyt-*c* + dATP)-mediated proteolytic processing. In SKLU-1 cells, the p37 caspase-9 cleaved forms was regularly detected in both the (cyt-*c* + dATP)-untreated and -treated cysosol samples.

**Figure 2.**
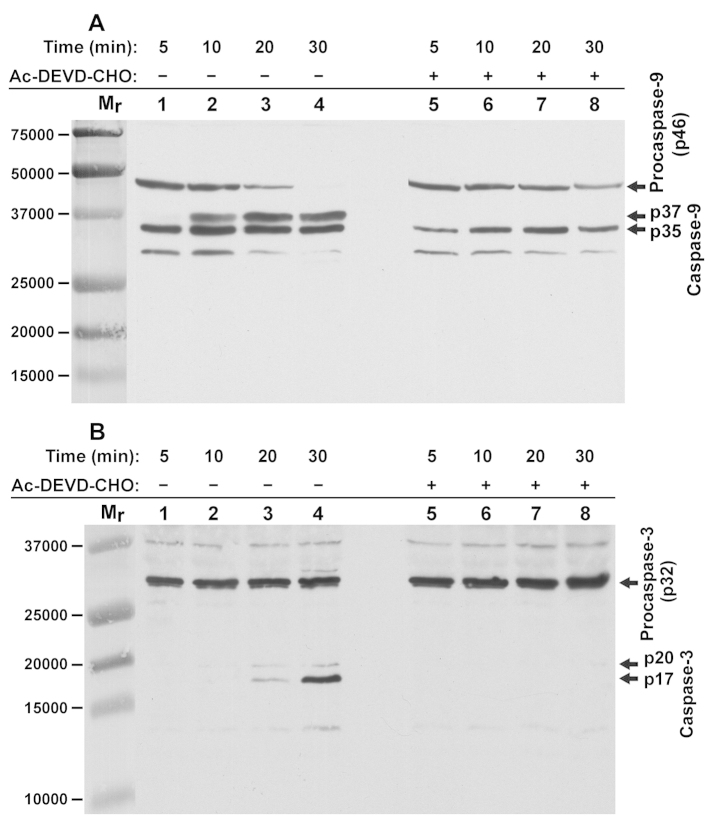
Analysis of the (cyt-*c* + dATP)-induced procaspase-9 processing and caspase-9 activity in NCI-H520 cell-free cytosol. In the absence of the caspase inhibitor Ac-DEVD-CHO, the (cyt-*c* + dATP)-induced processing of procaspase-9 (p46) to the p35 and p37 caspase-9 cleaved forms (A, left panel) was followed by procaspase-3 (p32) processing to the p20 and p17 caspase-3 cleaved forms (B, left panel). In the presence of Ac-DEVD-CHO (1 *μ*M), procaspase-9 (p46) was processed to the p35 caspase-9 cleaved form only (A, right panel) and the processing of procaspase-3 (p32) was completely blocked (B, right panel).

**Figure 3.**
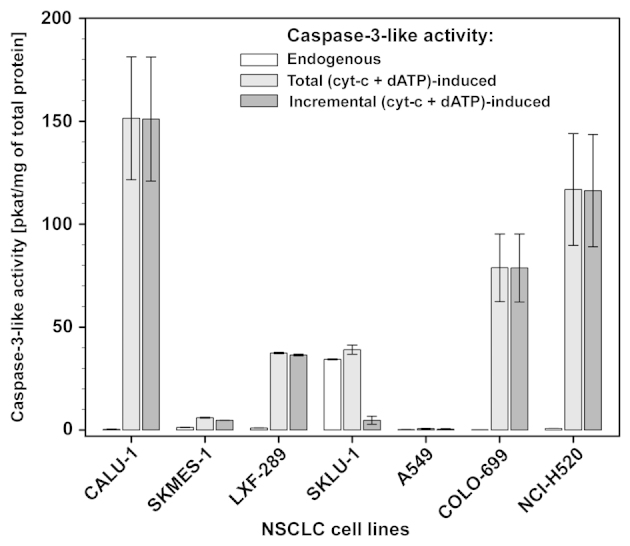
Analysis of the endogenous and the (cyt-*c* + dATP)-induced caspase-3-like activity in cell-free cytosols from NSCLC cell lines. The endogenous (E), the total (cyt-*c* + dATP)-induced (T), and the incremental (cyt-*c* + dATP)-induced (I=T–E) caspase-3-like activities were measured using Ac-DEVD-AFC as the substrate in cell-free cytosol samples from a panel of seven NSCLC cell lines. The cell-free cytosol from SKLU-1 cells regularly contained a high endogenous caspase-3-like activity. Data are indicated as mean ± SEM from three independent experiments.

It has been recently demonstrated that the activation of PC-9 at the Apaf-1-apoptosome requires its proteolytic processing to CS-9 (12). To assess the functionality of apoptosome apparatus in NSCLC cell lines, we studied the proteolytic processing of PC-9 to CS-9 and the subsequent proteolytic activation of PC-3 to CS-3 and generation of CS-3-like activity upon incubation of cell-free cytosol samples with exogenous cyt-*c* and dATP. In CALU-1, SKMES-1, LXF-289 and NCI-H520 cells, we detected the generation of the p35 CS-9 cleaved form already within 5–10 min after the addition of cyt-*c* and dATP, while the p37 CS-9 cleaved form appeared later (Figs. 1A and 2A, left part). Surprisingly, the p35 CS-9 form in the (cyt-*c* + dATP)-treated cell-free cytosol from LXF-289 cells, detected in 10 min of activation, disappeared later (Fig. 1). This may be due to the time-dependent and yet unexplained loss of an antigenic epitope from the C-terminus of the p35 caspase-9 subunit, which was recognised by the used anti-caspase-9 antibody. The (cyt-*c* + dATP)-treated cell-free cytosol from A549 cells showed PC-9 protein (p46), but the p35 and p37 cleaved CS-9 forms were not detected, indicating inhibition of apoptosome activation (Fig. 1B). Finally, the cell-free cytosol from SKLU-1 cells also did not respond with the p35 CS-9 form generation to the cyt-*c* and dATP treatment (Fig. 1B). However, we regularly detected a faint band of the p37 CS-9 form in the untreated as well as the (cyt-*c* + dATP)-treated cytosol samples from SKLU-1 cells (Fig. 1B), indicating the presence of an endogenous CS-3-like activity in these cells (Fig. 3).

To study the involvement of CS-3 in the proteolytic processing of PC-9, we incubated cell-free cytosol from NCI-H520 cells for different times with cyt-*c* and dATP and in the absence and the presence of a typical CS-3 inhibitor Ac-DEVD-CHO (1 *μ*M). The western blot (WB) analysis showed that in the absence of the inhibitor, PC-9 was processed into two forms, one containing the p35 subunit and the other one the p37 subunit (Fig. 2A, lanes 2–4). The formation of the CS-9 p35 form preceded the generation of the CS-9 p37 form (Fig. 2A, lane 1 vs. lane 2). The time-dependent disappearance of PC-9 (p46) was faster in samples without Ac-DEVD-CHO compared to the samples with Ac-DEVD-CHO (Fig. 2A, lanes 1–4 vs. lanes 5–8). In addition, the formation of the CS-9 p37 form correlated with the generation of active CS-3 forms containing the p20 or p17 subunit (Fig. 2A and B, left parts). On the contrary, the generation of both the CS-9 p37 form and the active CS-3 forms was completely blocked in the presence of Ac-DEVD-CHO (Fig. 2A and B, right parts). These results clearly indicate that Ac-DEVD-CHO can inhibit the activity of CS-9 p35 form in the holo-apoptosome, leading to the absence of PC-3 activation, but it does not inhibit the apoptosome-driven PC-9 activation and autoprocessing to CS-9 p35 form.

**Figure 4.**
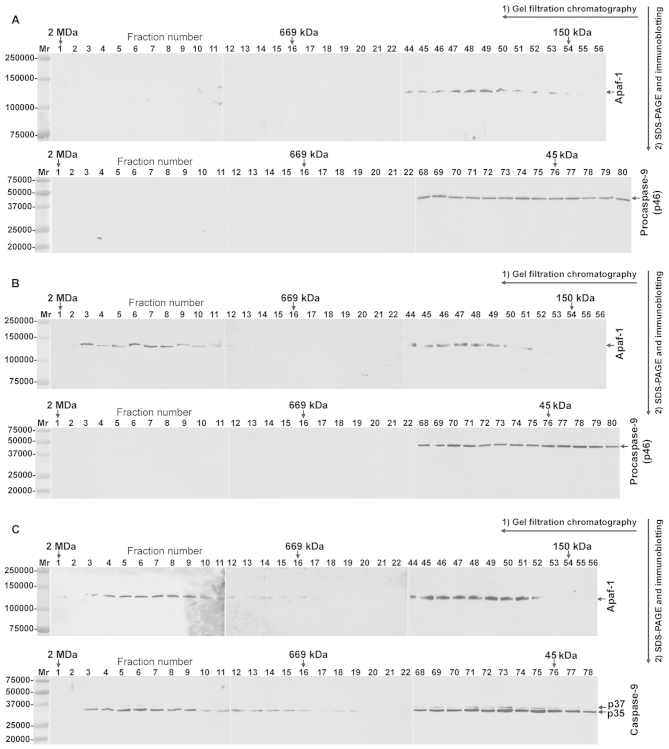
Analysis of the formation of apoptosome complexes in cell-free cytosol from COLO-699 cells. (A) Non-incubated control cell-free cytosol: both Apaf-1 and unprocessed procaspase-9 (p46) eluted in low M_r_-fractions. (B) Cell-free cytosol after incubation without exogenous cytochrome *c* and dATP: Apaf-1 eluted in both high and low M_r_-fractions, while procaspase-9 (p46) eluted in low M_r_-fractions only. (C) Cell-free cytosol after incubation with exogenous cytochrome *c* and dATP: Apaf-1 eluted in both high and low M_r_-fractions, while procaspase-9 (p46) was completely converted to the p35 caspase-9 cleaved form, which coeluted with Apaf-1 in high M_r_-fractions and also eluted in low M_r_-fractions. The positions of M_r_ markers are indicated with arrows. For further experimental details see Materials and methods.

To further confirm functionality or suppression of apoptosome apparatus in the studied NSCLC cell lines, we analysed and compared the levels of the endogenous and the (cyt-*c* + dATP)-induced CS-3-like activity in their cell-free cytosol, using a fluorimetric CS-3-like activity assay with Ac-DEVD-AFC as the substrate. The (cyt-*c* + dATP)-treated cytosol samples showed a significant increase of CS-3-like activity in 5 (CALU-1, NCI-H520, SKMES-1, LXF-289 and COLO-699) of 7 studied NSCLC cell lines (Fig. 3), confirming the results of WB analysis of the (cyt-*c* + dATP)-induced processing of PC-9 and PC-3 proteins (Figs. 1, 2A and 4C). The cell-free cytosol samples from A549 and SKLU-1 cells did not show any significant increase of the endogenous CS-3-like activity after the treatment with cyt-*c* and dATP (Fig. 3). Interestingly, we regularly found a high level of endogenous CS-3-like activity in the (cyt-*c* + dATP)-untreated cell-free cytosol from SKLU-1 cells (Fig. 3). This unusual finding was accompanied with the expression of a p24 protein co-detected by WB with the unprocessed PC-3 (unpublished data). Both, the endogenous and the (cyt-*c* + dATP)-induced CS-3-like activities were completely inhibited by the caspase inhibitor Ac-DEVD-CHO at 10 *μ*M (data not shown).

**Figure 5.**
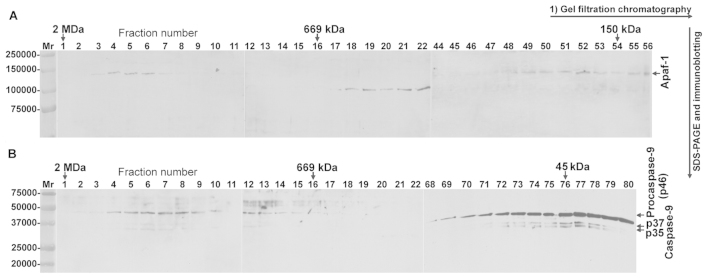
Analysis of the formation of apoptosome complexes in cell-free cytosol from A549 cells. After incubation of A549 cell-free cytosol with exogenous cytochrome *c* and dATP, Apaf-1 and procaspase-9 (p46) co-eluted in high-M_r_ fractions as well as in low-M_r_ fractions. Small amounts of p35 and p37 caspase-9 cleaved forms were found in low-M_r_ fractions. The positions of M_r_ markers are indicated with arrows. For further experimental details see Materials and methods.

To find out the possible assembly difference between the functional and dysfunctional apoptosomes in the studied NSCLC cell lines, we analysed the formation of apoptosome complexes in cell-free cytosol, using GFC and WB techniques. As the primary control, we used cell-free cytosol from NSCLC cell lines which was kept on ice, instead of being incubated prior to GFC. Using this material from COLO-699 cells, Apaf-1 and PC-9 eluted in M_r_-region corresponding to their unoligomerized forms (Fig. 4A). Surprisingly, if the cytosol from COLO-669 cells was incubated alone at 37°C for 30 min, Apaf-1, but not PC-9, eluted in two M_r_-regions, one corresponding to its high-M_r_ aggregate (∼1,400 kDa) and the other one to its unoligomerized forms (Fig. 4B, upper panel). On the contrary, when the cytosol from COLO-669 cells was incubated with cyt-*c* and dATP at 37°C for 30 min, PC-9 was completely converted to the CS-9 p35 and p37 cleaved forms, which co-eluted with Apaf-1 in two M_r_-regions, one corresponding to their high-M_r_ complexes (∼1,400 and ∼700 kDa) and the other one to their unoligomerized forms (Fig. 4C). Similar results were obtained in the apoptosome assembly experiments carried out with the cell-free cytosol from CALU-1 cells (data not shown). By contrast, the incubation of A549 cell-free cytosol with cyt-*c* and dATP induced a shift of a portion of both Apaf-1 (Fig. 5A) and PC-9 (Fig. 5B) proteins into the high-M_r_ fractions corresponding to complexes of ∼1,400 and ∼700 kDa. These fractions contained predominantly PC-9 while the CS-9 p35 and p37 forms were only barely detectable when using three times higher concentration of the anti-caspase-9 antibody. However, the majority of PC-9 and some CS-9 eluted in the low-M_r_ fractions (Fig. 5B).

**Figure 6.**
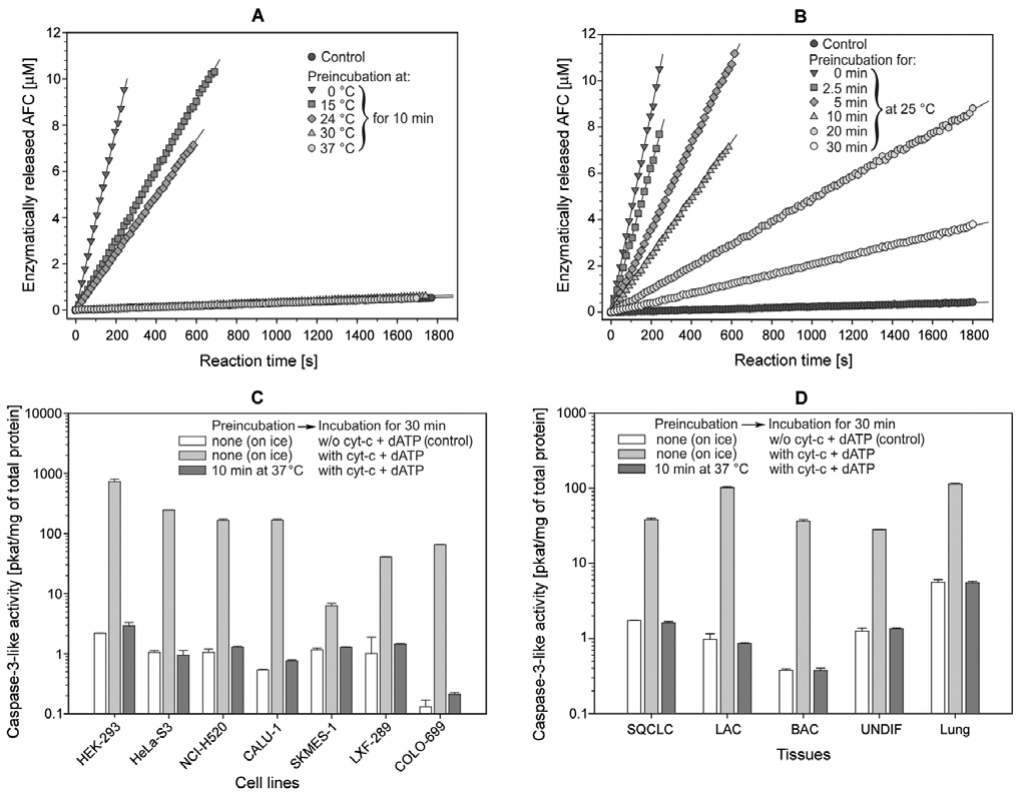
Thermal pre-treatment of cell-free cytosol causes loss of the (cyt-*c* + dATP)-mediated induction of caspase-3-like activity: dependency on temperature and time, and its effect in different cell lines and NSCLC tumours and lung parenchyma. Cell-free cytosol from NCI-H520 cells was pre-incubated without cyt-*c* and dATP at different temperatures (A) and for different times (B), following incubation with exogenous cyt-*c* and dATP at 37°C for 30 min. Cytosol samples without pre-incubation (kept on ice) that were incubated without and with cyt-*c* plus dATP served as negative and positive controls, respectively. Cell-free cytosols from different cell lines (C) and NSCLC tumours of different histopathological types and lung parenchyma (D) were kept on ice or were pre-incubated at 37°C for 10 min following incubation without or with cyt-*c* and dATP at 37°C for 30 min. After completing the indicated treatments, the endogenous and the total (cyt-*c* + dATP)-induced caspase-3-like activities were measured using Ac-DEVD-AFC as the substrate. One representative data set of three independent experiments is shown in (A) and (B). Data in (C) and (D) represent means ± SEM from three independent experiments for each examined cell line and tissue. Note the logarithmic scale on the y-axis in (C) and (D).

To asses the effect of thermal pre-treatment of the cell-free cytosol on the (cyt-*c* + dATP)-induced CS-3-like activity, we pre-incubated aliquots of the cytosol from NCI-H520 cells without cyt-*c* and dATP at different temperatures and for different times. Subsequently, the samples were incubated with exogenous cyt-*c* and dATP at 37°C for 30 min and then assayed, along with controls, for CS-3-like activity. As shown in Fig. 6A and B, the (cyt-*c* + dATP)-mediated induction of CS-3-like activity was rapidly lost in cytosol samples which were exposed to thermal pre-treatment during the period of apoptosome activation. Similar results were obtained in the thermal pre-treatment experiments carried out at different temperatures and for different times with the cell-free cytosol from CALU-1 cells (data not shown). Importantly, the pre-incubation at 37°C for 10 min leads to irreversible loss of apoptosome activation ability in all cell-free cytosol samples tested, including cytosols from seven cell lines (Fig. 6C), four NSCLC tumours of different histopathological types and lung parenchyma (Fig. 6D). These results indicate that thermal pre-treatment of cell-free cytosol samples should be avoided in apoptosome activation analysis in order to prevent loss of apoptosome activation ability.

We next studied the sensitivity of apoptosome apparatus to activation in NSCLC tumours and matched lungs, using analysis of CS-3-like activity in cell-free cytosols. The endogenous (E) as well as the (cyt-*c* + dATP)-induced total (T) and the (cyt-*c* + dATP)-induced incremental (I; I=T-E) CS-3-like activities were significantly higher in NSCLC tumours as compared to matched lungs (Table I). When the robust (cyt-*c* + dATP)-induced CS-3-like activity generation (i.e. I/E ≥2) was considered, it was found in 19 (31%) of 62 examined tumours and only in 5 (8%) of 62 examined lungs (p=0.003; χ^2^ test). When all (cyt-*c* + dATP)-dependent increases of CS-3-like activity were considered (i.e. I/E >0), they were found in 47 (76%) of 62 examined tumours and 29 (47%) of 62 examined lungs (p=0.0017; χ^2^ test). Moreover, the tumour/lung CS-3-like activity ratio was much higher than 2 for the (cyt-*c* + dATP)-induced activities (T and I) and was the predominant finding in the majority of examined NSCLC patients (Table I). These results indicate that the apoptosome apparatus can be more robustly and more frequently activated in NSCLC tumours as compared to matched lungs. Nevertheless, the tumour histopathological type, grade and stage or the patient’s gender and smoking habit had no statistically significant impact on the levels of endogenous and the (cyt-*c* + dATP)-induced CS-3-like activity in the tumours (p>0.08, Mann-Whitney test; data not shown).

**Figure 7.**
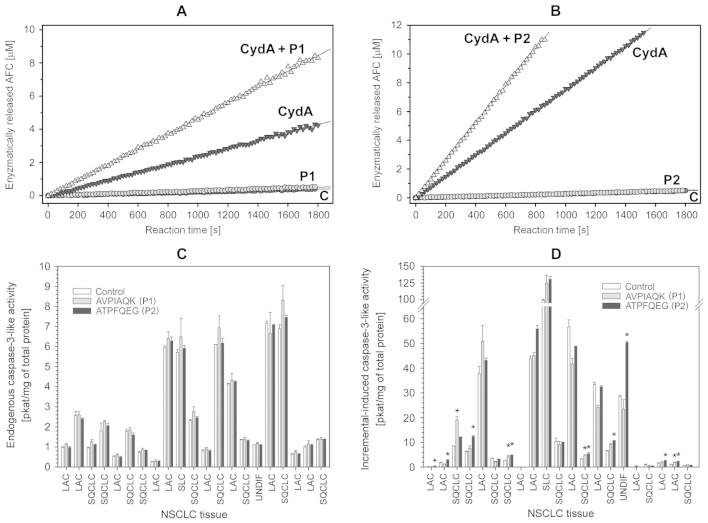
Effect of XIAP-neutralizing peptides on the endogenous and the (cyt-*c* + dATP)-induced caspase-3-like activity in cell-free cytosols from NSCLC tumours. (A and B) Caspase-3-like reactions in cell-free cytosol sample aliquots from an SQCLC tumour and a UNDIF tumour, respectively, that had been preincubated without (a control, C) or with cyt-*c* + dATP (CydA) and without or with 500 *μ*M of the XIAP-neutralizing peptide AVPIAQK (P1) or ATPFQEG (P2). In (A), the rates of the caspase-3-like reactions labelled C, P1, CydA and CydA + P1 were, respectively, 0.23, 0.29, 2.4 and 4.7 nkat. In (B), the rates of the caspase-3-like reactions labelled C, P2, CydA and CydA + P2 were, respectively, 0.28, 0.28, 7.6 and 13.0 nkat. (C and D) depict the responses of the endogenous and the (cyt-*c* + dATP)-induced caspase-3-like activities, respectively, in cell-free cytosols from 21 NSCLC tumours to the peptides AVPIAQK (P1, 500 *μ*M) and ATPFQEG (P2, 500 *μ*M). The incremental (cyt-*c* + dATP)-induced caspase-3-like activities in the absence of the peptides (a control, I) and in their presence (I-P1 and I-P2) were calculated as described in Materials and methods. Data indicated as mean ± SEM from three independent experiments. The caspase-3-like activity ratios I-P1/I and I-P2/I higher than 2 (+) and higher than 1.5 (*) but lower than 2 are indicated.

We further tested whether XIAP, the only direct and simultaneous inhibitor of CS-9 and CS-3 or CS-7 (42–45), inhibits the apoptosome activity in cytosol from NSCLC tumours. The levels of XIAP protein assayed in the cytosols from NSCLC tumours (n=28) ranged from 17.6 to 126.6 nmol per mg of total protein. The (cyt-*c* + dATP)-untreated cytosol samples from these tumours showed negative linear correlation between the level of XIAP protein and the low endogenous CS-3-like activity (r=−0.480, p=0.009). However, once treated with cyt-*c* and dATP, the cytosol samples displayed positive linear correlation between the level of XIAP protein and the (cyt-*c* + dATP)-induced CS-3-like activity (r=0.414, p=0.028). This indicates that the apoptosome-generated CS-3 activity can overcome the potential caspase inhibitory effect of XIAP in NSCLC tumours. To test whether XIAP is involved in the inhibition of endogenous and apoptosome-generated caspase activity in cytosol from NSCLC tumours we used the heptapeptides AVPIAQK (P1) and ATPFQEG (P2) to neutralize the XIAP inhibitory action against the caspases (38,46). In the (cyt-*c* + dATP)-untreated cytosol samples from NSCLC tumours (n=21), the respective endogenous CS-3-like activities (in pkat/mg of total protein, median and range) in the absence (E) and the presence of 500 *μ*M of peptide P1 (E-P1) or peptide P2 (EP-2) were: E, 1.36 and 0.27–7.16; EP-1, 1.39 and 0.28–8.30; and EP-2, 1.39 and 0.29–7.45. The slight increase of the endogenous CS-3-like activity in the presence of peptide P1 (Fig. 7A and C) was statistically significant (p=7.0×10^−4^, Wilcoxon matched pair test). When the cytosols samples from NSCLC tumours were treated with cyt-*c* and dATP in the presence of 500 *μ*M of peptide P1 or P2 some of them showed markedly higher CS-3-like activity as compared with the cytosol samples treated with cyt-*c* and dATP in the absence of the peptides (Fig. 7A, B and D). The respective (cyt-*c* + dATP)-induced incremental CS-3-like activities (in pkat/mg of total protein, median and range) in the absence (I) and the presence of 500 *μ*M of peptide P1 (I-P1) or peptide P2 (IP-2) were: I, 6.2 and 0.2–99.3; I-P1, 7.6 and 0.1–124.7; and I-P2, 9.9 and 0.4–130.5. The moderate increase of the (cyt-*c* + dATP)-induced incremental CS-3-like activity in the presence of peptide P2 was statistically significant (p= 0.012, Wilcoxon matched pair test). In addition, the CS-3-like activity ratios I-P1/I and I-P2/I higher than 1.5 were found in 4 (21%) and 9 (47%) of 19 evaluated NSCLC tumours, respectively (Fig. 7D), further indicating somewhat higher potency of peptide P2 to derepress the inhibition of apoptosome-generated CS-3-like activity.

## Discussion

The results of the present study showed that apoptosome apparatus was functional in the majority of examined NSCLC cell lines and NSCLC tumours. This evidence was obtained in experiments demonstrating the (cyt-*c* + dATP)-induced proteolytic processing of PC-9 to CS-9 leading to the proteolytic activation of PC-3 to CS-3 and to generation of CS-3-like activity in cell-free cytosol samples. Recent studies provided clear evidence that the activation of PC-9 at the Apaf-1-apoptosome requires its proteolytic processing to CS-9 (12). The lack of the (cyt-*c* + dATP)-mediated induction of CS-3-like activity we observed in cell-free cytosols from A549 and SKLU-1 cell lines was associated with the absence of PC-9 processing to the p35 CS-9 form.

It was previously reported that the ∼1,400- and ∼700-kDa apoptosome complexes, both containing the p35 and p37 forms of CS-9, were formed in the dATP-activated lysates from human tumour cells lines THP.1 and MCF-7 (13,14,33). In this study, we confirmed abundant formation of the high-M_r_ apoptosome complex of ∼1,400 kDa in the (cyt-*c* + dATP)-responsive COLO-699 and CALU-1 cytosols, but the ∼700 kDa complex was also detected. Interestingly, after treatment of the cytosol samples with cyt-*c* and dATP for 30 min, PC-9 was completely processed to CS-9 p35 and p37 forms. These CS-9 forms co-eluted with both apoptosome complexes, but were mostly present in the low-M_r_ fractions corresponding to CS-9 monomers. This can be explained by the recruitment-dependent PC-9 processing function of apoptosome, where PC-9 has higher binding affinity to apoptosome and displaces the processed CS-9 (10). Thus, in COLO-699 and CALU-1 cell-free cytosols, apparently all PC-9 molecules can be processed to CS-9 after the activation of apoptosome apparatus. By contrast, in the (cyt-*c* + dATP)-treated A549 cell-free cytosol, the formed high-M_r_ apoptosome complexes were able to bind PC-9, but the proteolytic processing of the apoptosome-associated PC-9 was blocked and the majority of PC-9 was present outside the apoptosomes. The mechanism underlying the failure of proteolytic processing of the apoptosome-bound PC-9 is not known at present. It might involve phosphorylations of PC-9 molecules that do not interfere with PC-9 recruitment to apoptosome, but can prevent the apoptosome-mediated PC-9 activation (48). The trace amounts of CS-9 p35 and p37 forms were probably extra-apoptosome generated CS-9 forms, since they could be immunodetected in A549 cell-free cytosol regardless of the treatment with cyt-*c* and dATP, using three times higher concentration of the anti-caspase-9 antibody (data not shown). Although the origin of the cleaved CS-9 forms in A549 cells cytosol is not known, for instance, calpains are capable of PC-9 cleavage to CS-9 p35 and p37 forms (47).

It was previously observed that incubation of recombinant Apaf-1 with cyt-*c* in the absence of dATP resulted in the formation of non-functional high-M_r_ Apaf-1 aggregates (34). In the present study, we found that incubating cell-free cytosol from all tested cell lines and tissues without addition of cyt-*c* and dATP at temperatures higher than 4°C leads to loss of the (cyt-*c* + dATP)-mediated induction of CS-3-like activity. This thermal-induced loss of apoptosome activation is likely due to not only the formation of high-M_r_ Apaf-1 aggregates incapable of recruitment and activation of PC-9 but also by yet unexplained inactivation of Apaf-1 monomers (Fig. 4B and C, upper panels). The Apaf-1 aggregates were not present in cell-free cytosols which were not exposed to thermal treatment prior to GFC and WB analysis (Fig. 4A upper panel). Moreover, contrary to a previous report (34), the addition of exogenous cyt-*c* during the thermal treatment of cell-free cytosols had a protective role on the subsequent (cyt-*c* + dATP)-induction of CS-3-like activity (data not shown). Our results indicate that the propensity of cytosolic Apaf-1 to irreversible thermal aggregation and inactivation complicates the attempts to reactivate non-functional apoptosomes, for instance through enzymatic dephosphorylation of phosphorylated PC-9 (48) or Apaf-1 (49,50).

Previous studies showed increased activation ability of apoptosomes in breast cancer cells (21) and brain tumours (22), because of PHAPI and Apaf-1 overexpression, respectively. In the present study, we found differential sensitivity to apoptosome apparatus activation in NSCLC tumours and matched lungs. The heightened sensitivity to apoptosome activation in these tumours may be caused by overexpression of both Apaf-1 and PC-3 proteins (27). Although the activation of apoptosome apparatus was much more robust and frequent in NSCLC tumours as compared to lungs, it was not associated with tumour histology, grade or stage. The low sensitivity to apoptosome activation of lung parenchyma cells, which are exposed to various environmental stressors, is important for their long-term survival and restriction from unwanted apoptosis. It has been already reported that compared to inflammatory cells airway epithelial cells have some innate resistance to apoptosis stimuli (51). Thus, the differential sensitivity of NSCLC tumours and lung parenchyma to apoptosome activation might be exploited for the apoptosis-based therapies. On the other hand, however, the apoptosome-generated CS-3 activity in tumour cells can significantly increase the rate of tumour recurrence and death of patients (23,24). The mechanism of this cancer cell repopulation involves the CS-3-mediated proteolytic activation of calcium independent phospholipase-A_2_ leading to increased production of arachidonic acid and prostaglandin-E_2_, which stimulates proliferation of tumour cells (23,24).

XIAP acts in the initiation as well as the execution phases of apoptosome pathway due to inhibition of the apoptosome-associated CS-9 p35/p12 form, CS-3 and CS-7 (42,44,46,52). Since XIAP is highly expressed in NSCLC tumours (40), it might be involved in suppression of apoptosome activity in these tumours. However, the inhibition of the apoptosome-associated CS-9 by XIAP can be reverted by Smac protein, which sequesters XIAP and relieves CS-9 to activate PC-3 to CS-3 (46). The active CS-3, but not CS-7, in turn removes the IAP binding motif from the N-terminus of CS-9 p12 subunit making the CS-9 resistant to XIAP inhibition (46). To test whether the inactivation of XIAP can enhance the activity of apoptosome apparatus in cytosol from NSCLC tumours, we used the XIAP-neutralizing peptides AVPIAQK (P1) and ATPFQEG (P2). These peptides displayed similar binding affinities toward XIAP as the target (46). The former peptide (38) as well as their shorter analogues (52) were previously shown to be very effective derepressors of the apoptosome aparatus. We found, that the (cyt-*c* + dATP)-induced CS-3-like activity was moderately increased by the CS-9 p12 subunit-mimetic peptide ATPFQEG in some tested tumours. These results indicate that XIAP does not effectively suppress the activity of the apoptosome apparatus in NSCLCs.

## Figures and Tables

**Table I.** Analysis of the (cytochrome *c* + dATP)-induced activation of apoptosome apparatus in cytosols from non-small cell lung carcinoma tissues and matched lung parenchyma.

Category of caspase-3-like activity	Caspase-3-like activity ^a,b^ (pkat/mg of total protein)		Statistical difference (p-value) of caspase-3-like activity in Tu vs. Lu^c^	Tu/Lu ratio of caspase-3-like activity^b,d^	No. of patients with Tu/Lu activity ratio >2

	Tumours (Tu)	Lungs (Lu)			
Endogenous (E)	0.717 (0–7.710)	0.019 (0–1.351)	9.2×10^−11^	13.8 (0.3–107.1)	27 (79%)
Total induced (T)	1.428 (0–88.706)	0.064 (0–5.645)	9.8×10^−11^	17.1 (0.6–773.3)	38 (88%)
Incremental induced (I)^e^	0.289 (0–88.429)	0.004 (0–5.245)	1.3×10^−5^	9.1 (0.3–2,364.9)	19 (70%)
				
Activity ratio I/E^f^	1.07 (0.03–536.3)	0.49 (0.001–28.4)			

a A total of 62 NSCLC patients was studied including 29 patients with SQCLC tumours, 26 patients with LAC tumours, 1 patient with SQCLC+LAC mixed type tumour, 1 patient with LCLC tumour, 3 patients with SLC tumours and 2 patients with UNDIF tumours.

b Data are represented as the median with the range in parentheses.

c Statistical difference of caspase-3-like activity in tumours versus lungs was calculated using Mann-Whitney test.

d Tumour/lung caspase-3-like activity ratio for the E, T and I activities could be calculated in 34, 43 and 27 NSCLC patients, respectively.

e Incremental induced activity was calculated as the difference between the total induced and the endogenous activities (I=T-E).

f The ratio I/E could be calculated in 47 tumours and 29 lungs. Chi-square test showed that the apoptosome apparatus was more frequently activated in tumour cytosols than in lung cytosols (p=0.0017).
